# Comparative Efficacy and Safety of Once-Weekly Pegylated Recombinant Human Growth Hormone Versus Daily Growth Hormone Therapy in Children: A Systematic Review and Meta-Analysis

**DOI:** 10.3390/jcm14248740

**Published:** 2025-12-10

**Authors:** Bassam Bin-Abbas, Mosleh Ali Jabari

**Affiliations:** 1 Department of Pediatrics, King Faisal Specialist Hospital and Research Center, Riyadh 11211, Saudi Arabia; 2 Department of Pediatrics, College of Medicine, Imam Mohammad Ibn Saud Islamic University (IMSIU), Riyadh 11564, Saudi Arabia

**Keywords:** growth hormone deficiency, idiopathic short stature, PEGylated recombinant human growth hormone, long-acting growth hormone, meta-analysis, pediatric endocrinology

## Abstract

**Background:** Childhood growth hormone deficiency (GHD) and idiopathic short stature (ISS) are endocrine disorders characterized by impaired linear growth due to insufficient or ineffective growth hormone (GH) activity. While daily recombinant human GH (rhGH) therapy effectively restores growth, treatment adherence remains suboptimal owing to the burden of daily injections. Long-acting formulations such as pegylated recombinant human GH (PEG-rhGH) have been developed to improve convenience and compliance while maintaining therapeutic efficacy. This systematic review and meta-analysis aimed to evaluate the comparative effectiveness and safety of once-weekly PEG-rhGH versus daily rhGH and to assess dose–response outcomes between higher- and lower-dose PEG-rhGH regimens in pediatric GHD and ISS. **Methods:** This study followed PRISMA 2020 guidelines. Comprehensive searches were conducted in PubMed, Web of Science, and Scopus from inception to September 2025 using MeSH terms and free-text keywords for “PEGylated recombinant human growth hormone,” “long-acting growth hormone,” and “growth hormone deficiency.” Eligible studies included randomized controlled trials (RCTs) and cohort studies evaluating PEG-rhGH in children (≤18 years) with GHD or ISS, comparing either once-weekly PEG-rhGH with daily rhGH or different PEG-rhGH doses. Data extraction included study design, participant characteristics, intervention details, and key outcomes (height SDS, height velocity, IGF-1 SDS). Meta-analysis was conducted using Review Manager with a random-effects model, and heterogeneity was quantified using the I^2^ statistic. **Results:** Eight studies, comprising 2549 children, met the inclusion criteria. Once-weekly PEG-rhGH demonstrated comparable short-term growth outcomes to daily rhGH at 6 and 12 months, with modest but significant superiority in height SDS (MD = 0.10, 95% CI 0.01–0.19) and height velocity (MD = 0.74 cm/year, 95% CI 0.42–1.05) by 24 months. IGF-1 SDS did not differ significantly at 6 or 12 months. In dose comparisons, 0.2 mg/kg/week PEG-rhGH produced substantially greater gains in height SDS and IGF-1 SDS than 0.1 mg/kg/week, with a time-dependent increase in the magnitude of the effect. Safety analyses revealed no increase in adverse or serious adverse events with PEG-rhGH compared to daily rhGH; reactions were generally mild and transient. **Conclusions:** Once-weekly PEG-rhGH is as effective as daily rhGH for promoting growth in pediatric GHD and ISS, with possible long-term advantages in growth outcomes and similar safety. The higher PEG-rhGH dose (0.2 mg/kg/week) appears to optimize efficacy without compromising tolerability. Weekly administration may enhance adherence and quality of life, supporting PEG-rhGH as a viable alternative to daily GH therapy.

## 1. Introduction

Childhood growth hormone deficiency (GHD) is an endocrine disorder characterized by reduced production or activity of growth hormone (GH), resulting in impaired linear growth, delayed skeletal maturation, and short stature relative to age- and sex-matched peers. The condition can present as a singular pituitary shortage or as part of many pituitary hormone irregularities, with an estimated prevalence of 1 in 4000 to 10,000 children [1,2]. If inadequately addressed, GHD not only inhibits growth but also alters body composition, bone density, lipid metabolism, and mental and social well-being [3]. Idiopathic short stature (ISS) denotes a condition characterized by an individual’s height being more than 2 standard deviations below the mean height for their specific age, gender, and demographic, without an identifiable underlying cause. Children with ISS have normal birth size, appropriate body proportions, adequate nutritional intake, absence of behavioral illnesses, and no indications of GHD or chromosomal abnormalities [4]. Short stature occurs in approximately 2.3% of children [1]. In nearly 70% of cases evaluated for short stature, children appear healthy and show no clinical or laboratory abnormalities to explain their growth delay; these cases are typically classified as constitutional short stature or ISS [5].

Since the introduction of recombinant human growth hormone (rhGH) therapy in 1985, daily subcutaneous rhGH injections have served as the primary treatment for childhood GHD and ISS, exhibiting well-documented efficacy in restoring near-normal growth velocity and attaining final adult height [6,7]. GH therapy is also widely utilized in other growth-limiting conditions, including Turner syndrome, small for gestational age (SGA) without catch-up growth, and particularly in patients with SHOX gene haploinsufficiency—one of the most common monogenic causes of short stature, where GH treatment has demonstrated superior growth responses [8,9]. It is important to note that, unlike GHD, GH therapy for ISS is not universally accepted or approved across countries. Regulatory policies vary substantially, and in several regions, GH treatment for ISS is not authorized for routine clinical use. Despite its efficacy, adherence to regular rhGH administration remains a significant challenge. Studies consistently demonstrate that 30–50% of pediatric patients exhibit partial or inadequate adherence due to the inconvenience and discomfort associated with daily injections [10,11]. Noncompliance with regulations has been associated with reduced stature, diminished insulin-like growth factor 1 (IGF-1) responses, and increased healthcare expenditures [12]. Facilitating treatment adherence has been a primary focus in the management of GHD and ISS.

To address this desire, long-acting growth hormone (LAGH) formulations have been developed to reduce the frequency of injections while maintaining stable endogenous GH levels. These preparations utilize several techniques, including pegylation, depot formulations, prodrug designs, and fusion proteins [13]. Pegylated recombinant human growth hormone (PEG-rhGH) is among the earliest and most extensively studied LAGH molecules. The covalent bonding of PEG extends the half-life of GH, stabilizes its structure, and reduces its renal clearance rate. This indicates that GH and IGF-1 can remain effective during weekly administrations [14,15].

This systematic review and meta-analysis aimed to compare (1) once-weekly PEG-rhGH with daily rhGH and (2) higher-dose versus lower-dose PEG-rhGH regimens in pediatric GHD and ISS. This analysis seeks to elucidate the clinical efficacy and dose optimization of PEG-rhGH therapy by including the most recent data, thereby promoting more individualized and adherence-enhancing treatment methods for children with GHD and ISS.

## 2. Materials and Methods

### 2.1. Search Strategy

This systematic review and meta-analysis was conducted in accordance with the PRISMA 2020 guidelines [16] (Supplementary Material). A comprehensive literature search was conducted in the PubMed, Web of Science, and Scopus databases from their inception to September 2025. The search combined controlled vocabulary (MeSH and Emtree terms) and free-text keywords related to GHD and long-acting pegylated formulations. The search terms included: “Pegylated growth hormone” OR “PEG-rhGH,” OR “PEGylated recombinant human growth hormone” OR “Long-acting growth hormone” OR “Weekly growth hormone” AND “Growth hormone deficiency” OR “Idiopathic short stature” AND “Children” OR “Pediatric.” Reference lists of all included studies, relevant reviews, and clinical trial registries (ClinicalTrials.gov, WHO ICTRP) were screened for additional eligible publications.

### 2.2. Eligibility Criteria

Studies were eligible if they met the following criteria:

1. Population: Pediatric patients (≤18 years) diagnosed with GHD or ISS.
2. Intervention: Once-weekly PEG-rhGH administered at any dose.
3. Comparator: Either conventional once-daily rhGH or a different PEG-rhGH dosing regimen.
4. Outcomes: Studies reporting at least one relevant outcome, including height SDS, height velocity (cm/year), or IGF-1 SDS.
5. Design: Randomized controlled trials (RCTs), prospective non-randomized controlled studies, or retrospective cohort studies with a control or comparison group.

Exclusion criteria were:

- Adult populations (>18 years).
- Non-pegylated long-acting GH analogs (e.g., somapacitan, or somatrogon).
- Case reports, reviews, or conference abstracts without full data.
- Duplicate publications from overlapping cohorts.

### 2.3. Study Selection and Screening

All titles and abstracts were screened for relevance using Rayyan (https://www.rayyan.ai/, accessed on 15 September 2025) [17]. Potentially eligible articles were retrieved in full text and reviewed for inclusion. The selection process was summarized in a PRISMA flow diagram outlining the numbers identified, screened, excluded, and included.

### 2.4. Data Extraction

A standardized extraction sheet was used to collect data on: Study characteristics (author, year, design, sample size, and follow-up duration), participant characteristics (age, sex, and weight), intervention details (PEG-rhGH dose, frequency, and comparator regimen), and outcomes (height SDS, height velocity, and IGF-1 SDS).

### 2.5. Quality Assessment and Risk of Bias

The methodological quality of RCTs was evaluated using the Cochrane Risk of Bias 2.0 (RoB 2) tool [18], which assesses potential bias across five domains: the randomization process, deviations from intended interventions, missing outcome data, outcome measurement, and selective reporting. For observational studies, quality appraisal was performed using the Newcastle–Ottawa Scale (NOS) [19], which evaluates methodological soundness across three domains—selection of study groups, comparability of cohorts, and assessment of outcomes—with a maximum attainable score of 9. Studies achieving a score of 7 or higher were classified as high-quality.

### 2.6. Statistical Analysis

Meta-analyses were conducted when at least two studies provided comparable data for a given outcome, using Review Manager (RevMan) software (version 5.4) [20]. The mean difference (MD) with corresponding 95% confidence intervals (CI) was used as the summary statistic for continuous outcomes, and statistical significance was defined as *p* < 0.05. When means or standard deviations (SDs) were not directly reported, they were derived from alternative summary statistics following established methods. Changes from baseline values were extracted preferentially over final values; when the SD of change was unavailable, it was estimated using reported *p*-values and an assumed correlation coefficient of 0.75. If baseline data were missing, outcome values were used instead. When data were presented graphically, numerical values were extracted using the Plot Digitizer (https://plotdigitizer.com/ (accessed on 25 September 2025)) online tool [21]. A random-effects model (inverse-variance method) was applied to account for potential inter-study variability, providing more conservative and generalizable pooled estimates than a fixed-effects approach. Statistical heterogeneity was assessed using the chi-square test (*p* ≤ 0.10) and quantified using the I^2^ statistic, where values exceeding 40% indicated moderate to high heterogeneity. Greater emphasis was placed on I^2^ values, as they offer a more reliable measure when the number of studies is limited. Subgroup analyses were conducted according to different follow-up time points for each outcome. Owing to the small number of included studies, publication bias could not be formally evaluated using funnel plots.

## 3. Results

### 3.1. Searching and Screening

**Figure 1:**
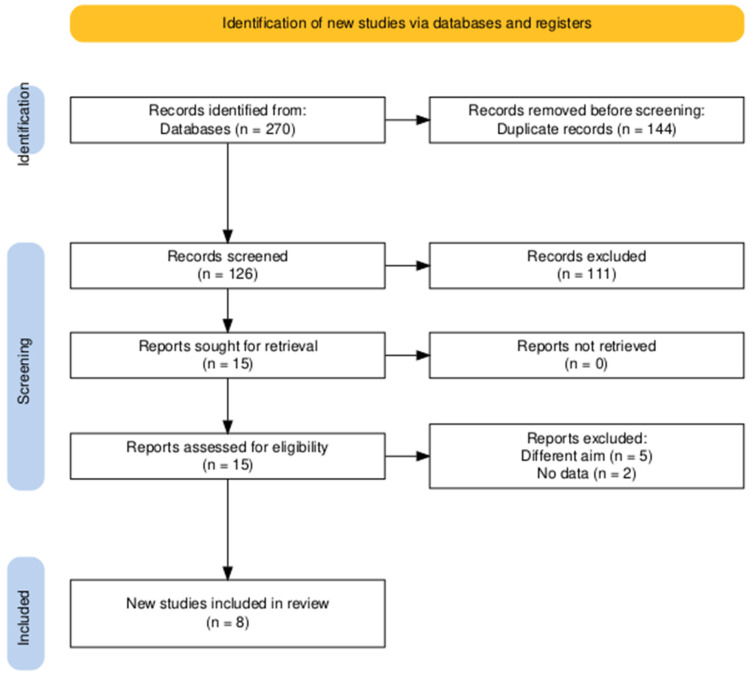
PRISMA flow diagram of searching and screening.

Our searching process in the assigned databases resulted in a total of 270 articles. After duplicate removal, title and abstract screening was conducted on 126 articles. Of the 126 records screened by title and abstract, 111 were excluded due to wrong population (*n* = 42), wrong intervention (*n* = 27), wrong outcomes (*n* = 31), non-original or non-comparative designs such as reviews or case reports (*n* = 9), and duplicate data from overlapping cohorts (*n* = 2). This process yielded 15 articles for full-text review, and eight of them [13,22,23,24,25,26,27,28] were eligible for final inclusion (Figure 1).

### 3.2. Characteristics of the Studies

Eight studies encompassing six RCTs and 3 cohort studies (*n* = 2549) met the inclusion criteria: five studies comparing once-weekly PEG-rhGH with once-daily GH and four studies directly comparing higher- versus lower-dose PEG-rhGH. Across studies, the mean chronological age at baseline was 7.4 years. Interventions fell into two comparisons: (i) dose comparison of once-weekly PEG-rhGH 0.2 vs. 0.1 mg/kg/week and (ii) head-to-head efficacy of once-weekly PEG-rhGH (typically 0.14–0.20 mg/kg/week) versus conventional once-daily rhGH. Follow-up assessments were most commonly reported at 1, 3, 6 and 12 months; two studies extended to 24 months. A detailed summary of the characteristics of the included studies is shown in Table 1.

### 3.3. Quality Assessment and Risk of Bias

**Figure 2:**
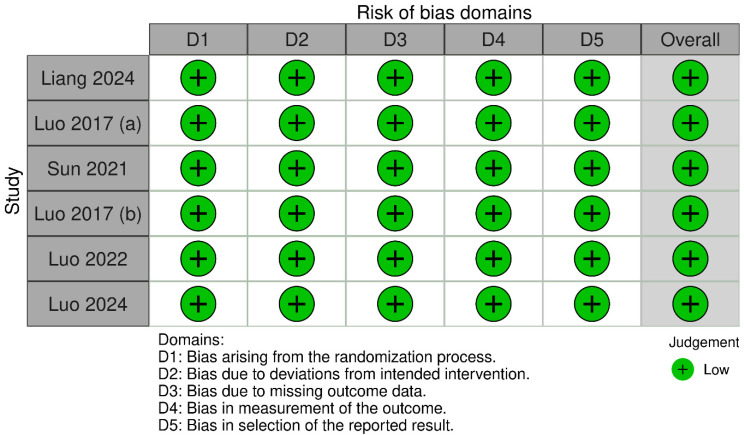
Risk of bias assessment of RCTs using RoB 2 [22,23,24,25,27].

According to RoB 2, the six RCTs were deemed to have low risk of bias across all domains (Figure 2). Regarding the cohort studies assessed with NOS, the three studies were observed to have high quality (Table 2).

### 3.4. Statistical Analysis

#### 3.4.1. Long-Acting PEG-rhGH vs. Daily GH

**Figure 3:**
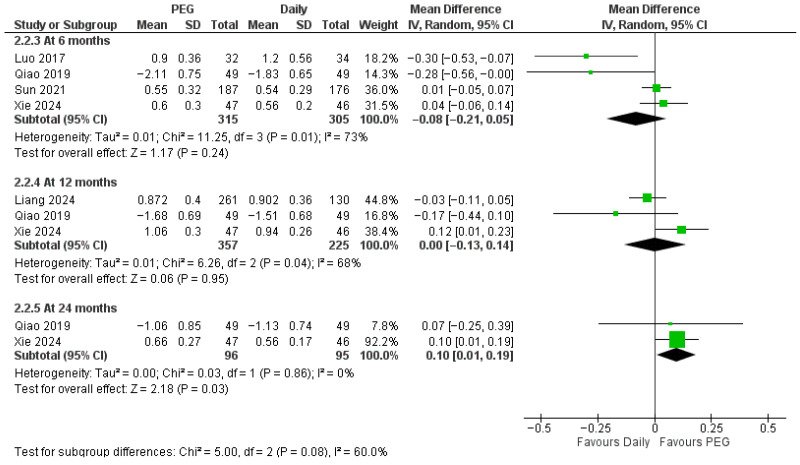
Comparison between long-acting PEG-rhGH and daily GH in height SDS [22,23,26,27,28].

When weekly PEG-rhGH was compared with daily rhGH, short-term efficacy in growth outcomes was similar, but advantages for PEG-rhGH emerged at longer durations. For height SDS (Figure 3), groups were comparable through 6 and 12 months (MD = −0.08 SDS, 95% CI −0.21 to 0.05 and MD = 0 SDS, −0.13 to 0.14, respectively), while a weakly significant advantage for PEG-rhGH appeared by 24 months (MD = 0.10 SDS, 95% CI 0.01 to 0.19). There were significant subgroup differences (*p* = 0.08).

**Figure 4:**
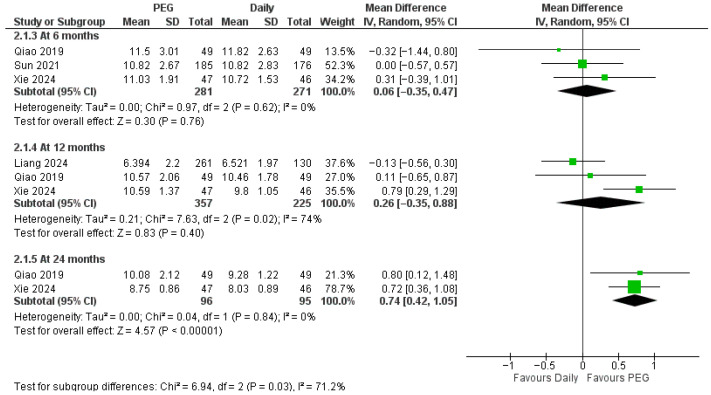
Comparison between long-acting PEG-rhGH and daily GH in height velocity [22,26,27,28].

Height velocity showed a similar pattern (Figure 4): no difference at 6 months (MD = 0.06 cm/year, 95% CI −0.35 to 0.47) or 12 months (MD = 0.26 cm/year, 95% CI −0.35 to 0.88) but a large significant benefit for PEG-rhGH at 24 months (MD = 0.74 cm/year, 95% CI 0.42 to 1.05; subgroup differences were significant, *p* = 0.03).

**Figure 5:**
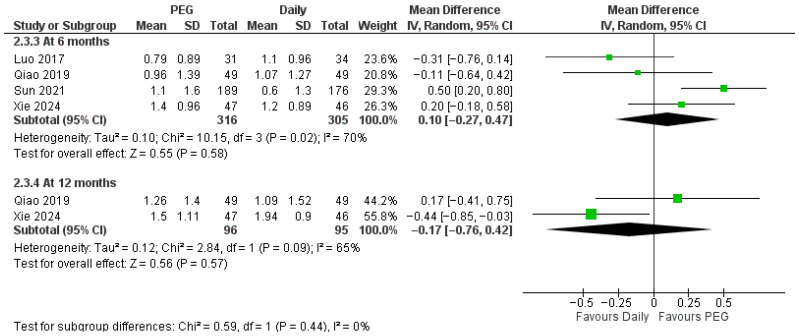
Comparison between long-acting PEG-rhGH and daily GH in IGF-1 SDS [23,26,27,28].

Regarding IGF-1 SDS (Figure 5), pooled effects showed no significant difference at 6 months (MD = 0.10 SDS; 95% CI −0.27 to 0.47) or 12 months (MD = −0.17 SDS; 95% CI −0.76 to 0.42), and there was no subgroup interaction by time (*p* = 0.44). Collectively, weekly PEG-rhGH provides growth outcomes comparable to those of daily rhGH in the first year and may confer incremental advantages in linear growth velocity and height SDS by the second year.

#### 3.4.2. Dose Comparison (PEG-rhGH 0.2 vs. 0.1 mg/kg/week)

**Figure 6:**
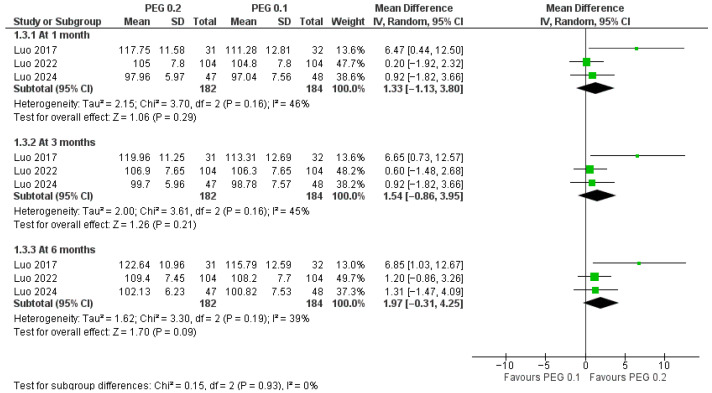
Comparison between PEG-rhGH 0.2 and 0.1 mg/kg/week in height [23,24,25].

Higher-dose PEG-rhGH produced consistent advantages over 0.1 mg/kg/week across growth and biochemical outcomes, and the magnitude of these advantages generally increased with time on treatment. For linear growth in centimeters (Figure 6), pooled mean differences (MDs) favored 0.2 mg/kg/week at 1 month (MD = 1.33 cm; *p* = 0.29; I^2^ = 46%), 3 months (MD = 1.54 cm; *p* = 0.21; I^2^ = 45%), and 6 months (MD = 1.97 cm, *p* = 0.09; I^2^ = 39%) but without statistical significance. The between-time subgroup test was not significant (*p* = 0.93), indicating stable effects across different time windows.

**Figure 7:**
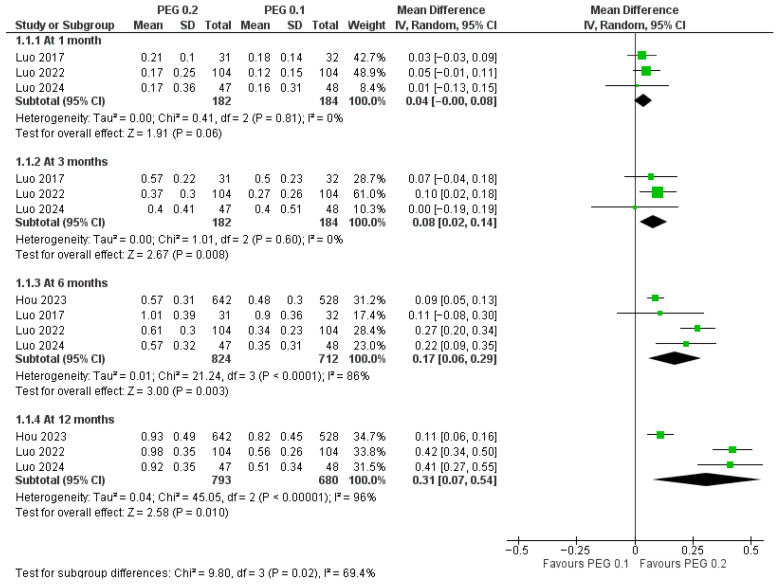
Comparison between PEG-rhGH 0.2 and 0.1 mg/kg/week in height SDS [13,23,24,25].

These centimeter-scale gains translated into statistically significant improvements in height SDS from 3 months onward (Figure 7): MD = 0.04 SDS (*p* = 0.06; I^2^ = 0%) at 1 month; 0.08 SDS (*p* = 0.008; I^2^ = 0%) at 3 months; 0.17 SDS (*p* = 0.003; I^2^ = 86%) at 6 months; and 0.31 SDS (*p* = 0.01; I^2^ = 96%) at 12 months. Although heterogeneity increased at later time points, the direction of the effect remained uniformly in favor of the higher dose, and the test for subgroup differences by time was significant (*p* = 0.02), indicating an increase in the effect size over time.

**Figure 8:**
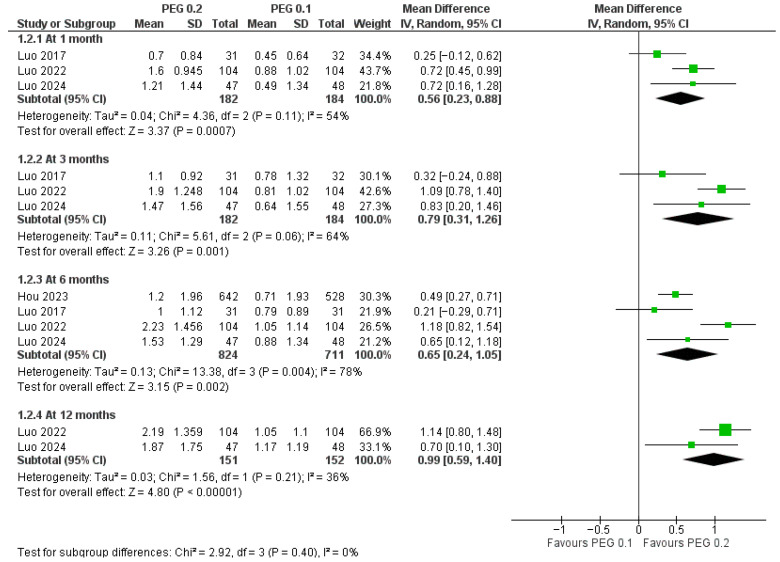
Comparison between PEG-rhGH 0.2 and 0.1 mg/kg/week in IGF-1 SDS [13,23,24,25].

Biochemically, IGF-1 SDS increased more with 0.2 mg/kg/week at every assessment (Figure 8): MD = 0.56 SDS (*p* = 0.0007; I^2^ = 54%) at 1 month, 0.79 SDS (*p* = 0.001; I^2^ = 64%) at 3 months, 0.65 SDS (*p* = 0.002; I^2^ = 78%) at 6 months, and 0.99 SDS (*p* < 0.00001; I^2^ = 36%) at 12 months; there was no evidence that the IGF-1 SDS effect varied by time (*p* = 0.40).

Taken together, these findings demonstrate a clinically coherent pattern in which the higher weekly PEG-rhGH dose yields larger, time-accumulating gains in both Height SDS and IGF-1 SDS, with centimeter-scale growth advantages already evident by 3–6 months.

Safety analyses showed that the overall adverse event profile of PEG-rhGH was similar to that of daily rhGH, with no significant increase in serious or treatment-limiting complications. Reported adverse effects were primarily mild and transient, including localized injection-site reactions and asymptomatic increases in IGF-1, aligning with established safety data for long-acting GH analogs [25,27,28].

## 4. Discussion

### 4.1. Summary of Findings

This meta-analysis pooled data from eight clinical studies involving 2549 children with GHD or ISS and provides the most comprehensive quantitative synthesis to date, evaluating both the comparative efficacy of once-weekly PEG-rhGH versus conventional daily rhGH and the dose–response effects within PEG-rhGH regimens. The findings demonstrate that once-weekly PEG-rhGH achieved comparable short-term growth outcomes to daily rhGH over the first 12 months, with potential incremental advantages emerging over longer treatment durations. Specifically, PEG-rhGH produced modest but statistically significant gains in SDS and height velocity by 24 months of therapy, suggesting a sustained anabolic effect associated with improved treatment adherence and stable GH exposure profiles. In contrast, early IGF-1 SDS responses did not differ significantly between regimens, indicating biochemical equivalence during the first year of treatment.

In the dose-comparison analysis, higher-dose PEG-rhGH (0.2 mg/kg/week) consistently outperformed the lower-dose regimen (0.1 mg/kg/week) across all growth and biochemical indices, with effect sizes increasing over time. The higher dose produced significantly greater improvements in height SDS and IGF-1 SDS beginning at 3 months and persisting through 12 months, while linear height velocity gains reached clinical relevance by 6 months. Importantly, these advantages accumulated without evidence of disproportionate heterogeneity or loss of effect consistency across time points, implying a genuine dose-dependent relationship. Collectively, the findings reinforce that PEG-rhGH at 0.2 mg/kg/week provides optimal growth promotion, striking a balance between efficacy and safety.

The safety profile was similar between the two groups, regardless of whether they received PEG-rhGH or the daily regimen. These results support the clinical non-inferiority and practical advantages of weekly PEG-rhGH relative to daily injections, suggesting that reduced injection frequency may improve long-term adherence and, consequently, growth outcomes.

### 4.2. Investigation with Prior Literature

Multiple trials have now shown that weekly PEG-GH matches or exceeds the growth efficacy of daily GH in children. Likewise, a recent Phase III RCT found the once-weekly “Pegpesen” GH (140 μg/kg/week) to be non-inferior to daily GH (245 μg/kg/week) for 1-year growth velocity and height SDS, with similar safety and adherence [22]. These findings align with a broader consensus that long-acting GH formulations (pegylated or otherwise) generally produce non-inferior annual height velocities and comparable safety to daily GH in pediatric GHD [29].

Studies in non-GHD short stature also support the efficacy of weekly PEG-GH. In ISS, a phase II RCT reported dose-dependent improvements: at 52 weeks the high-dose PEG-GH (0.2 mg/kg/week) produced a mean Δheight SDS of +0.98 (vs +0.20 in controls) and significantly higher height velocity and IGF-1 SDS [24]. In a 2-year real-world Chinese cohort, children on weekly PEG-GH (0.2–0.3 mg/kg/week) had significantly greater catch-up growth than those on daily GH (mean Δheight SDS +1.65 vs. +1.50, *p* = 0.001) and higher annual height velocities (10.6 vs. 9.8 cm/year in year 1) [28]. Treatment adherence was also markedly better on the weekly regimen in that study (fewer missed doses). Similarly, in small children born small for gestational age (SGA), a 1-year trial showed that weekly PEG-GH (0.2 mg/kg/week) increased height SDS by +0.92 and height velocity to 9.94 cm/year, significantly more than a lower dose [25]. In Turner syndrome, a retrospective series found that girls receiving high-dose PEG-GH (0.2 mg/kg/week) achieved Δheight SDS and height velocity comparable to those on daily GH (0.38 mg/kg/week), both of which exceeded the outcomes of a lower-dose group [30]. Across these varied etiologies, IGF-1 levels rose on therapy but generally remained within normal range (only a few patients briefly exceeded +3 SDS) [30].

Safety profiles were analogous between weekly and daily regimens. Total adverse event rates (e.g., edema, headache, arthralgia, and hypothyroidism) did not differ significantly in trials [25,27,28]. Injection-site reactions were infrequent and mild. Importantly, no new safety signals emerged with PEG-GH: no treatment-related serious adverse events or diabetes cases were reported, and no patients developed GH antibodies in these studies [30]. In summary, the literature indicates that once-weekly PEG-GH can achieve growth outcomes at least as good as daily GH, with similar safety, while offering improved adherence in practice.

### 4.3. Clinical Implications

Regarding dosing and monitoring, weekly PEG-GH is typically initiated at the label dose (for example, Jintrolong 0.2–0.3 mg/kg/week for GHD or ISS) and titrated by weight and IGF-1 response [24,31]. Patients should have their serum IGF-1 level measured approximately 4 days after injection to estimate the average level of IGF-1. Doses are adjusted to maintain IGF-1 in the mid-normal range (approximately 0 SDS); if IGF-1 exceeds 2 SD, doses may be reduced (per consensus practice). Routine pediatric GH monitoring involves tracking growth velocity, bone age, thyroid function, and glucose metabolism, as well as screening for scoliosis or changes in head circumference as needed. Practitioners should note that IGF-1 kinetics differ with weekly GH (higher peak and trough); careful timing of lab draws is essential, as outlined in guidelines [31].

With regard to the benefits of weekly therapy, the chief advantage of PEG-GH is the reduced injection burden. Fewer injections substantially decrease treatment burden and may improve quality of life [32]. Caregivers and patients overwhelmingly prefer weekly injections due to their convenience. This can translate into better adherence: for example, one study reported PEG-GH patients missed a mean of only 0.75 doses/year versus 4.4 for daily GH over 2 years. Better compliance may lead to optimal height outcomes. Reduced injection frequency also minimizes pain, anxiety and clinic visits, which is especially beneficial for children with injection fear or complex social circumstances [31].

Regarding patient-reported outcomes, although formal quality of life comparisons are limited, available evidence suggests similar psychosocial benefits with weekly versus daily GH administration. A recent review found no detriment to quality of life on long-acting GH [32], and consensus experts anticipate improved well-being with less frequent dosing. Clinicians should engage patients/families in shared decision-making, weighing individual preferences and lifestyle when choosing weekly vs. daily regimens [31].

Considering the health system and policy, access to PEG-GH varies by region. In China, weekly PEG-GH (Jintrolong) is approved for GHD, ISS, and SGA. In other markets, long-acting GH analogs (e.g., somatrogon and lonapegsomatropin) are FDA/EMA-approved for GHD, but PEG-GH per se may not be available yet. Cost is a key consideration: LAGH products have higher unit prices than daily GH. However, no published cost-effectiveness analyses are yet available [32]. Payers may require demonstration of inadequate adherence or specific diagnostic criteria for coverage. Nonetheless, if long-term gains and reduced healthcare visits materialize, weekly therapy could be economically justified. Large registry studies are underway (for example, the China CGLS cohort of 10,000 short-stature patients) to compare the long-term safety, height outcomes, and healthcare utilization of PEG-GH versus daily GH [33]. These real-world data, together with emerging international experience, will inform guidelines and policy.

Clinicians considering PEG-GH should follow updated consensus guidance: start with weight-based dosing, monitor IGF-1 and growth as with daily GH, and adjust therapy to reach treatment goals. Counseling on injection technique and the importance of adherence remains crucial. In practice, weekly PEG-GH offers a viable alternative to daily injections, particularly for patients struggling with daily therapy, providing comparable efficacy with potential improvements in adherence and quality of life.

### 4.4. Strengths and Limitations

Strengths of this meta-analysis include a comprehensive synthesis of all available trials in children, encompassing multiple etiologies of short stature and incorporating both randomized controlled trials and real-world data. The total sample (2549 children) provides a robust basis for conclusions about growth outcomes. By analyzing both weekly vs. daily comparisons and dose-ranging studies, we capture important clinical questions. However, some limitations exist. The designs varied (RCTs and cohorts), and follow-up times ranged from 6 months to 3 years, introducing heterogeneity. Few studies extended beyond 2 years, so very long-term adult height outcomes remain uncertain. Additionally, publication bias is a possibility, as negative studies may be underreported. Some studies were led by the same research group, raising the possibility of partial overlap in participant cohorts across different publications. Such duplication could artificially inflate sample size or introduce bias in pooled estimates. However, we ensured that these were different samples by thorough checking of the methods to avoid this bias and inflation. All the included studies classified GHD based on stimulation testing but did not provide detailed etiological breakdowns (congenital vs. acquired GHD). Importantly, none of the studies explicitly reported inclusion of patients with GHD secondary to sellar or suprasellar tumors (such as craniopharyngioma) or post-surgical pituitary lesions. Likewise, data on tumor recurrence or monitoring were not presented in any of the included trials. Therefore, the safety profile observed here should not be extrapolated to children receiving GH after brain tumor treatment, where separate clinical considerations apply. All included studies were conducted in China. Although GH pharmacodynamics are generally similar across ethnicities, the exclusive representation of Chinese pediatric populations may limit external generalizability to other ethnic groups, such as White, Black, and Middle Eastern populations. Additional multinational trials are needed to validate these findings in more diverse cohorts.

### 4.5. Recommendations

Based on current evidence, once-weekly PEG-rhGH (especially at 0.2 mg/kg/week) appears to be an effective and safe treatment for pediatric short stature, with the convenience of fewer injections and better adherence. Clinicians may consider this regimen for eligible patients who may benefit from a reduced injection burden, provided the cost and availability are appropriate. Future research should focus on diverse populations to confirm applicability, and on long-term outcomes such as adult height and metabolic effects. Additional head-to-head trials comparing PEG-rhGH with other long-acting GH formulations would be valuable. Investigation into optimal dosing schedules (individualized adjustments) and monitoring strategies for IGF-1 is also warranted. Finally, the impact on patient quality of life and cost-effectiveness of weekly versus daily GH therapies should be evaluated.

## 5. Conclusions

This systematic review and meta-analysis indicated that once-weekly PEG-rhGH therapy is at least as effective as daily rhGH for promoting growth in children with short stature and may confer advantages in height velocity and SDS gain over longer treatment durations. The higher PEG-rhGH dose (0.2 mg/kg/week) consistently yielded greater improvements than the lower dose. Weekly PEG-rhGH was well tolerated, with no significant increase in adverse events compared to daily GH. These findings support the use of PEG-rhGH as a viable alternative to daily GH, particularly when adherence or injection frequency is a concern. Further studies are needed to verify these benefits in broader populations and to determine long-term outcomes.

## Figures and Tables

**Table 1:** Baseline characteristics of the included studies.

Study ID	Design	Intervention	Control	Sample Size		Chronological Age (Years), Mean (SD)		Male, *n* (%)		Weight (kg), Mean (SD)	
				PEG	Control	PEG	Control	PEG	Control	PEG	Control
Liang 2024 [22]	RCT	PEG-rhGH 140 μg/kg/week	Daily rhGH 245 μg/kg/week	261	130	6.68 (2.11)	7.03 (2.2)	172 (65.9)	87 (66.9)	18.46 (4.92)	18.83 (4.75)
Luo 2017 (a) [23]	RCT	PEG-rhGH 0.1 mg/kg/week	Daily rhGH 0.25 mg/kg/week	32	34	10.91 (3.31)	10.54 (4.05)	23 (71.88)	23 (67.65)	20.5 (7.23)	20.18 (6.06)
Qiao 2019 [26]	Cohort	PEG-rhGH 0.2 mg/kg/week	Daily rhGH 0.3 mg/kg/week	49	49	5.41 (2.37)	6.25 (2.42)	26 (53.1)	33 (67.3)	NR	NR
Sun 2021 [27]	RCT	PEG-rhGH 0.20 mg/kg	Daily rhGH 0.25 mg/kg	187	176	7.8 (2.8)	8 (2.9)	135 (72.2)	122 (69.5)	20.7 (7.1)	20.4 (6.1)
Xie 2024 [28]	Cohort	PEG-rhGH 0.2 mg/kg/week	Daily rhGH 0.38 mg/kg/week	47	48	6.6 (2.60	7.3 (1.8)	29 (61.7)	35 (72.9)	NR	NR
Hou 2023 [13]	Cohort	PEG-rhGH 0.2 mg/kg/week	PEG-rhGH 0.1 mg/kg/week	642	528	7.32 (2.56)	7.17 (2.54)	416 (64.8)	340 (64.39)	19.4 9(6.10)	19.4 (6.07)
Luo 2017 (b) [23]	RCT	PEG-rhGH 0.2 mg/kg/week	PEG-rhGH 0.1 mg/kg/week	31	32	11.75 (3.95)	10.91 (3.31)	25 (80.65)	23 (71.88)	23.01 (7.01)	20.5 (7.23)
Luo 2022 [24]	RCT	PEG-rhGH 0.2 mg/kg/week	PEG-rhGH 0.1 mg/kg/week	104	104	5.3 (1.29)	5.3 (1.34)	63 (60.6)	65 (62.5)	16.44 (2.93)	16.37 (2.52)
Luo 2024 [25]	RCT	PEG-rhGH 0.2 mg/kg/week	PEG-rhGH 0.1 mg/kg/week	47	48	4.3 (0.87)	4.3 (1.03)	24 (51.1)	29 (60.4)	13.43 (2.02)	13.18 (2.65)

RCT: randomized controlled trial, PEG-rhGH: recombinant human growth hormone, rhGH: recombinant human growth hormone, SD: standard deviation, NR: not reported, mg: milligrams, μg: microgram, kg: kilograms.

**Table 2:** Quality assessment of cohort studies using NOS. ☆ indicates one point awarded for each satisfied criterion according to the Newcastle–Ottawa Scale (NOS). The total number of stars reflects overall methodological quality, with a maximum score of 9 stars (4 for Selection, 2 for Comparability, and 3 for Outcome).

Study ID	Selection (Max 4)	Comparability (Max 2)	Outcome (Max 3)	Total (Max 9)
Qiao 2019 [26]	☆☆☆☆	☆☆	☆☆☆	☆☆☆☆☆☆☆☆☆
Xie 2024 [28]	☆☆☆☆	☆☆	☆☆☆	☆☆☆☆☆☆☆☆☆
Hou 2023 [13]	☆☆☆	☆☆	☆☆☆	☆☆☆☆☆☆☆☆

## Data Availability

The data supporting the findings of this study are available within the article and its Supplementary Materials. All datasets analyzed during the current meta-analysis were obtained from previously published studies, which are cited in the reference list.

## Supplementary Materials

The following supporting information can be downloaded at: https://www.mdpi.com/article/10.3390/jcm14248740/s1: PRISMA 2020 checklist.
